# A compact Cas9 ortholog from *Staphylococcus Auricularis* (SauriCas9) expands the DNA targeting scope

**DOI:** 10.1371/journal.pbio.3000686

**Published:** 2020-03-30

**Authors:** Ziying Hu, Shuai Wang, Chengdong Zhang, Ning Gao, Miaomiao Li, Deqian Wang, Daqi Wang, Dong Liu, Huihui Liu, Sang-Ging Ong, Hongyan Wang, Yongming Wang

**Affiliations:** 1 Obstetrics and Gynecology Hospital, State Key Laboratory of Genetic Engineering at School of Life Sciences, Zhongshan Hospital, Fudan University, Shanghai, China; 2 School of Life Sciences, Co-innovation Center of Neuroregeneration, Jiangsu Key Laboratory of Neuroregeneration, Nantong University, Nantong, China; 3 Experimental Center of Forestry in North China, Chinese Academy of Forestry, Beijing, China; 4 Department of Pharmacology, University of Illinois College of Medicine, Chicago, Illinois, United States of America; 5 Division of Cardiology, Department of Medicine, University of Illinois College of Medicine, Chicago, Illinois, United States of America; 6 Shanghai Engineering Research Center of Industrial Microorganisms, Shanghai, China; IMBA, AUSTRIA

## Abstract

Compact CRISPR/Cas9 systems that can be packaged into an adeno-associated virus (AAV) hold great promise for gene therapy. Unfortunately, currently available small Cas9 nucleases either display low activity or require a long protospacer adjacent motif (PAM) sequence, limiting their extensive applications. Here, we screened a panel of Cas9 nucleases and identified a small Cas9 ortholog from *Staphylococcus auricularis* (SauriCas9), which recognizes a simple NNGG PAM, displays high activity for genome editing, and is compact enough to be packaged into an AAV for genome editing. Moreover, the conversion of adenine and cytosine bases can be achieved by fusing SauriCas9 to the cytidine and adenine deaminase. Therefore, SauriCas9 holds great potential for both basic research and clinical applications.

## Abstract 2

A small ortholog of Cas9 isolated from Staphylococcus auricularis (SauriCas9), which recognizes a simple 5'-NNGG-3' protospacer adjacent motif (PAM), displays high activity for genome editing and is compact enough to be packaged into adeno-associated virus for genome editing.

## Introduction

The RNA-guided CRISPR/Cas9 system is a powerful tool for genome editing in diverse organisms and cell types [1–5]. In this system, a Cas9 nuclease and a guide RNA (gRNA) form a Cas9-gRNA complex, which recognizes a gRNA complementary DNA sequence and generates a site-specific double-strand break (DSB) [1,2,6]. Target site recognition also requires a specific protospacer adjacent motif (PAM) [6], which limits the targeting scope of Cas9 for precise positioning. Cas9 derived from *Streptococcus pyogenes* (SpCas9) is the most extensively applied variant because of its high efficiency and simple PAM requirement [6], but the SpCas9 gene (4.1 kbp) and its gRNA sequence are too large to be packaged together into an adeno-associated virus (AAV) [7] for efficient delivery into cells in vivo. In a search for smaller Cas9 nucleases, a type II-A *Staphylococcus aureus* Cas9 (SaCas9) was identified for in vivo genome editing [8], but this Cas9 ortholog is used infrequently because of the requirement of a longer PAM sequence (NNGRRT). Several small types of II-C Cas9 orthologs have been discovered for genome editing [9–13], but they either require long PAM sequence or display reduced activity [12,14,15]. Therefore, there remains a need for smaller Cas9 nucleases with high efficiency and shorter PAMs.

We have recently developed a highly sensitive green fluorescent protein (GFP) reporter–based method that allows PAM profiling in mammalian cells [16]. Here, we demonstrate that this method also enables the identification of Cas9 nucleases for genome editing in mammalian cells. Our newly developed method differs from previous screens, which typically rely on library cleavage in vitro or performed in bacteria in which identified Cas9 orthologs often do not work in mammalian cells [8,17]. We identified a naturally occurring Cas9 nuclease from *S*. *auricularis* (SauriCas9) that recognizes NNGG PAM, which occurs, on average, once in every 8 randomly chosen genomic loci. Importantly, SauriCas9 possesses a small gene size (3.3 kbp) that can be packaged into AAV together with its gRNA, holding great promise for gene therapy.

## Results

### Identification of SauriCas9 as a novel genome editing tool

**Fig 1:**
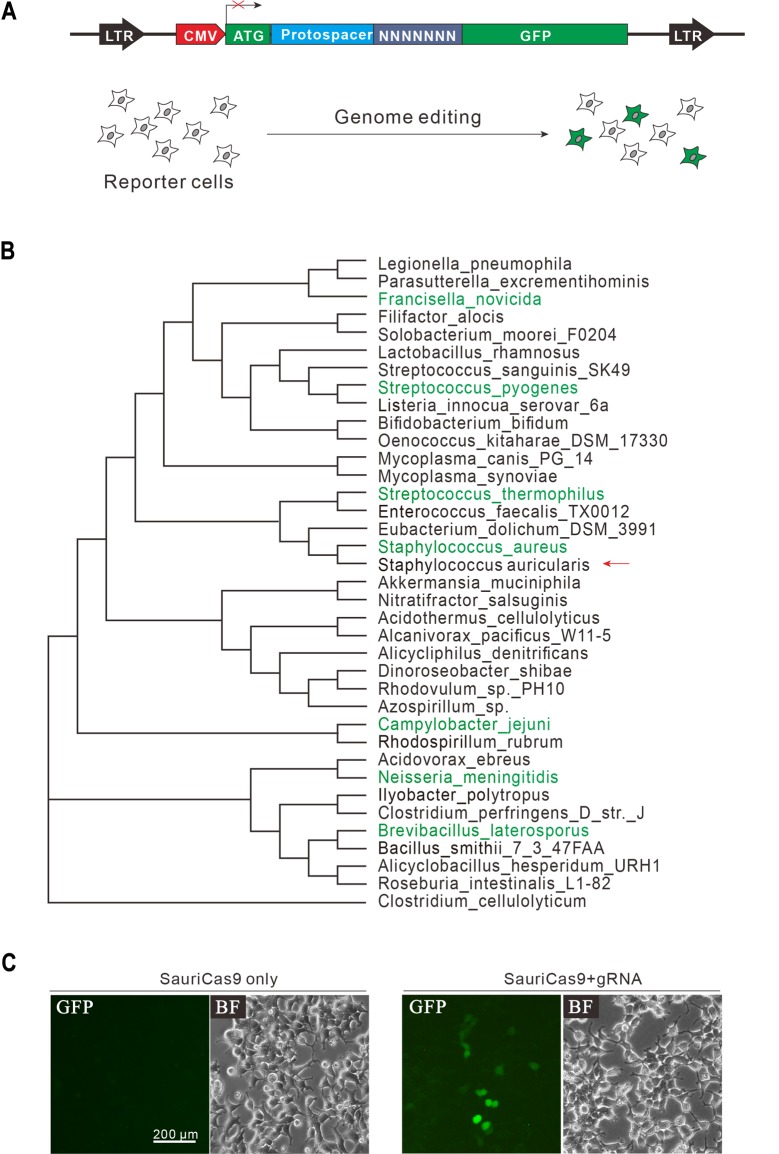
A GFP-reporter assay for Cas9 screening. (A) Schematic diagram of the GFP-reporter assay. A lentiviral vector containing a CMV-driven GFP, which is disrupted by an insertion of a target sequence followed by 7-bp random sequence between ATG and GFP coding sequence. The library DNA is stably integrated into HEK293T cells. Genome editing will induce GFP expression for a portion of cells. (B) Phylogenetic tree of selected Cas9 orthologs from different bacterial strains for activity screening. Seven validated Cas9 orthologs (green color) are used as reference. (C) Transfection of SauriCas9 with gRNA resulted in GFP expression, whereas transfection of SauriCas9 alone did not induce GFP expression. BF, bright field; CMV, cytomegalovirus; GFP, green fluorescent protein; gRNA, guide RNA; LTR, long terminal repeat; SauriCas9, Cas9 nuclease from *S*. *auricularis*.

To identify novel Cas9 nucleases for genome editing, we employed a GFP reporter–based method that allows Cas9 screening in mammalian cells [16]. In this method, GFP is inactivated by insertion of a target sequence followed by 7 bp of randomized DNA sequences. If a Cas9 ortholog facilitates DNA cleavage, transfection of it together with a gRNA will result in GFP expression (Fig 1A). We screened a panel of 30 Cas9 nucleases from different bacteria strains (Fig 1B). Each Cas9 gene was human codon optimized and cloned into a SaCas9 expressing vector by replacing SaCas9 [8]. The trans-activating CRISPR RNA (tracrRNA) sequences used were predicted by a bioinformatic tool (S1 Table) [18]. We designed gRNA scaffolds for each ortholog by fusing the 3′ end of a direct repeat with the 5′ end of the corresponding tracrRNA, including the full-length tail, via a 4-nucleotide linker. However, we did not observe any GFP-positive cells from the first round of screening. It was possible that we did not design proper gRNA scaffolds, leading to an unsuccessful screen. As it has been reported that tracrRNA can be exchanged between closely related CRISPR/Cas9 systems [19] and we noticed that SauriCas9 was phylogenetically related to SaCas9, we therefore combined SauriCas9 with SaCas9 tracrRNA, which led to GFP expression (Fig 1C), demonstrating the potential of this Cas9 nuclease for genome editing.

### PAM analysis

**Fig 2:**
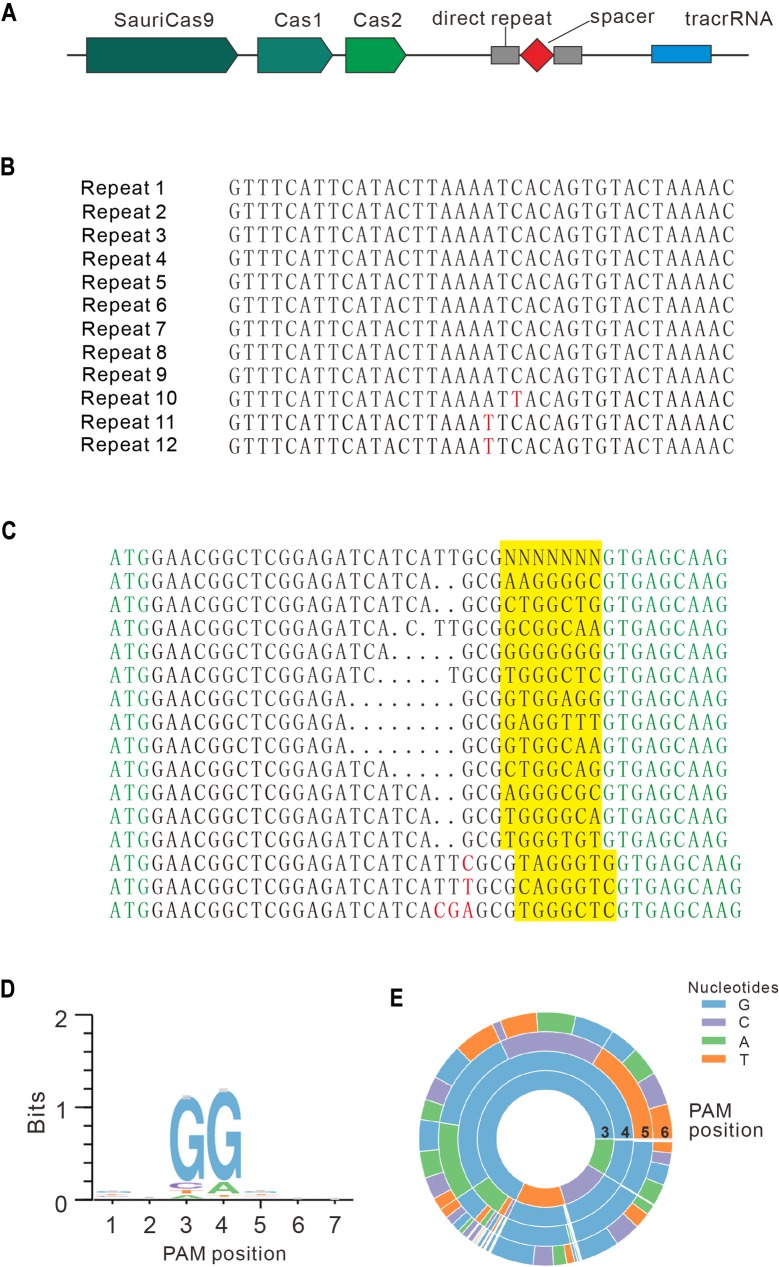
PAM sequence analysis for SauriCas9. (A) Genetic locus of CRISPR/SauriCas9. (B) Twelve CRISPR array repeat sequences were identified in CRISPR/SauriCas9 locus. Nucleotide mutations are shown in red. (C) Deep sequencing revealed that targets with NNGG PAM can be efficiently edited in the GFP-reporter assay. GFP sequence is shown in green; insertion mutations are shown in red; NNGG PAM sequences are highlighted in yellow; GCG trinucleotide is used to fix 7-bp random sequence. (D) WebLogo is generated from deep-sequencing data. (E) PAM wheel is generated from deep-sequencing data. GFP, green fluorescent protein; PAM, protospacer adjacent motif; SauriCas9, Cas9 nuclease from *S*. *auricularis*.

The CRISPR/SauriCas9 locus consists of 3 Cas genes, including Cas9, Cas1, and Cas2; 12 repeat sequences; and a putative tracrRNA (Fig 2A and 2B). Protein sequence alignment revealed that SauriCas9 (1,061 amino acids [aas]) has 62.4% sequence identity with SaCas9 (S1 Fig). We noticed that the SauriCas9 PAM-interacting domain (PID) has multiple aa variations compared with SaCas9 (S1 Fig). Importantly, the variations include 2 aas (S991 and L996) that are crucial for PAM recognition (S1 Fig) [20], suggesting that SauriCas9 may recognize PAMs differently from SaCas9. To identify PAM sequences that are recognized by SauriCas9, we sorted out GFP-positive cells, and sequences containing 7-bp randomized DNA were PCR amplified for deep sequencing. Sequencing results revealed that insertions/deletions (indels) were mainly associated with NNGG PAM (Fig 2C). Consistently, both WebLogo (http://weblogo.threeplusone.com/) and PAM wheel revealed that SauriCas9 preferred NNGG PAM (Fig 2D and 2E). In addition, PAM wheel revealed that SauriCas9 also recognized NNNGG PAM (Fig 2E).

### Genome editing with SauriCas9

**Fig 3:**
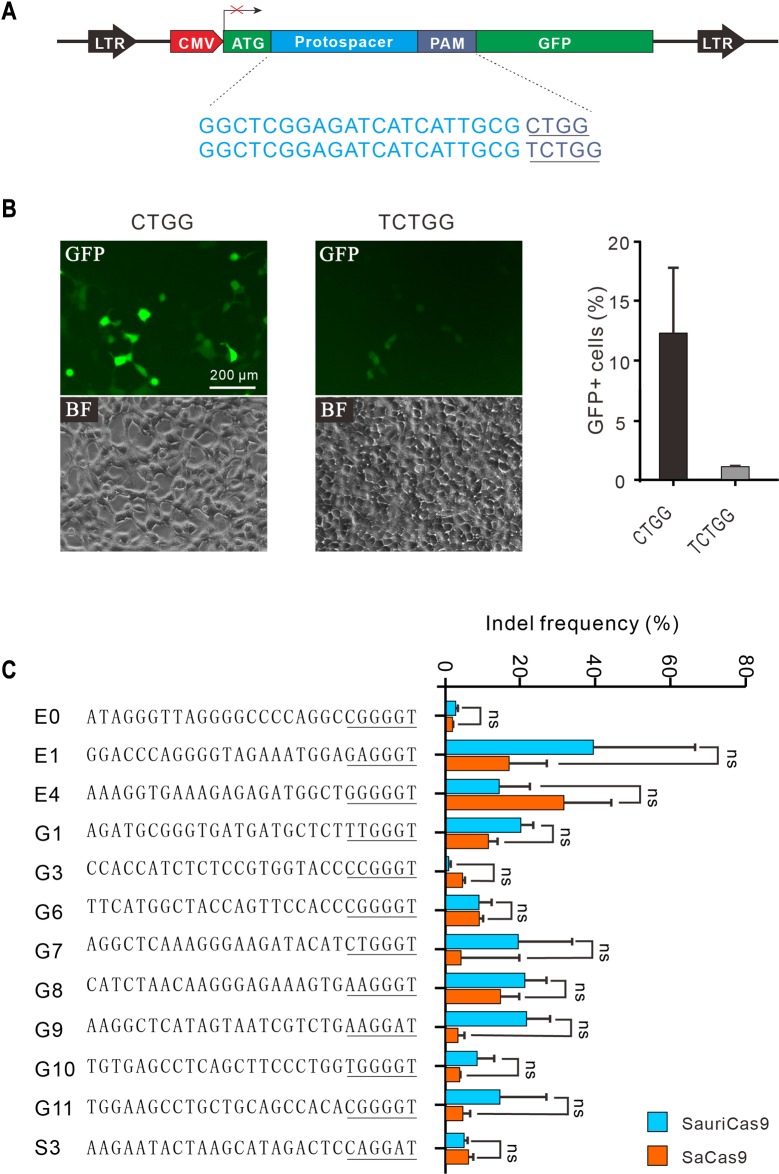
Genome editing capability of SauriCas9. (A) A GFP-reporter construct is used to compare the activity of NNGG and NNNGG PAMs. Target sequences are shown below. PAM sequences are shown in blue. (B) Transfection of SauriCas9 with gRNA resulted in GFP expression. Quantification is shown on the right. Underlying data for all summary statistics can be found in S1 Data. (C) Genome editing with SauriCas9 and SaCas9 for 12 endogenous loci. PAMs are underlined (*n* ≥ 2). Underlying data for all summary statistics can be found in S1 Data. GFP, green fluorescent protein; gRNA, guide RNA; PAM, protospacer adjacent motif; SaCas9, *S*. *aureus* Cas9; SauriCas9, Cas9 nuclease from *S*. *auricularis*.

To test the genome editing capability of SauriCas9, we then constructed GFP-reporter plasmids containing either NNGG or NNNGG (Fig 3A) and established stable cell lines expressing these reporters. Transfection of SauriCas9 with gRNA induced GFP expression for both PAMs, but the efficacy of NNNGG PAM was much lower than NNGG PAM (Fig 3B).

Next, we tested the genome editing capability of SauriCas9 with a panel of 12 endogenous gene targets in HEK293T cells. SauriCas9 generated indels at all 12 endogenous loci, but efficiencies varied depending on the targets (Fig 3C). These 12 target sites also contained NNGRRT PAM that can be edited by SaCas9, allowing a side-by-side comparison. Quantitative reverse transcription polymerase chain reaction (qRT-PCR) revealed that the expression of SauriCas9 and SaCas9 was comparable (S2 Fig). Although not significant, SauriCas9 showed higher activity at most of the tested loci (Fig 3C). We further tested genome editing capacity of SauriCas9 in additional cell types, including A375, A549, HeLa, human foreskin fibroblast (HFF, primary cells) cells, and N2a (mouse neuroblastoma cell line) cells. SauriCas9 generated indels in all these cell types with varying efficacies (S3A–S3E Fig). We also tested 3 endogenous targets with NNNGG PAM, but the indel frequencies were less than 2% (S4 Fig). We finally packaged SauriCas9 together with gRNA into AAV and infected either HEK293T, HCT116, A375, or HFF cells. Indels could be detected in all cell types, but frequencies varied depending on the loci and cell types (S5A–S5E Fig). Taken together, SauriCas9 offers a novel platform for genome editing.

### Base editing with SauriCas9

Base editing is a powerful technology that enables programmable conversion of single nucleotides in the mammalian genome. This technology relies on fusion of a catalytically disabled Cas9 nuclease to a nucleobase deaminase enzyme. Two types of programmable deaminases have been developed: cytosine base editors (CBEs), inducing C-to-T conversions [21], and adenine base editors (ABEs), inducing A-to-G conversions [22]. To increase base editing scope, several CRISPR nucleases have been employed, including SpCas9 [21,22], engineered SpCas9 variants [23,24], SaCas9 [24], and Cpf1 [25].

To test whether SauriCas9 can be employed for base editing, we generated nickase form of SauriCas9 (SauriCas9n) by introducing D15A mutation (S1 Fig). To confirm that SauriCas9n can induce nicks in genomic DNA, we inserted a pair of target sequences (E1 and S6) into a GFP-reporter plasmid and established a stable cell line (S6A Fig). If SauriCas9n is able to generate double nicking, indels will occur, and GFP-positive cells can be observed. The double-nicking strategy has been used to improve specificity of SpCas9 [26]. When we transfected wild-type SauriCas9 with a single gRNA, GFP expression could be easily observed (S6B Fig). When we transfected SauriCas9n with a single gRNA, GFP expression rarely occurred. However, when we transfected SauriCas9n with 2 gRNAs (E1 + S6) targeting each DNA strand, GFP expression could be easily observed, indicating that double nicking occurred. These data demonstrated that SauriCas9n is able to induce nicks.

**Fig 4:**
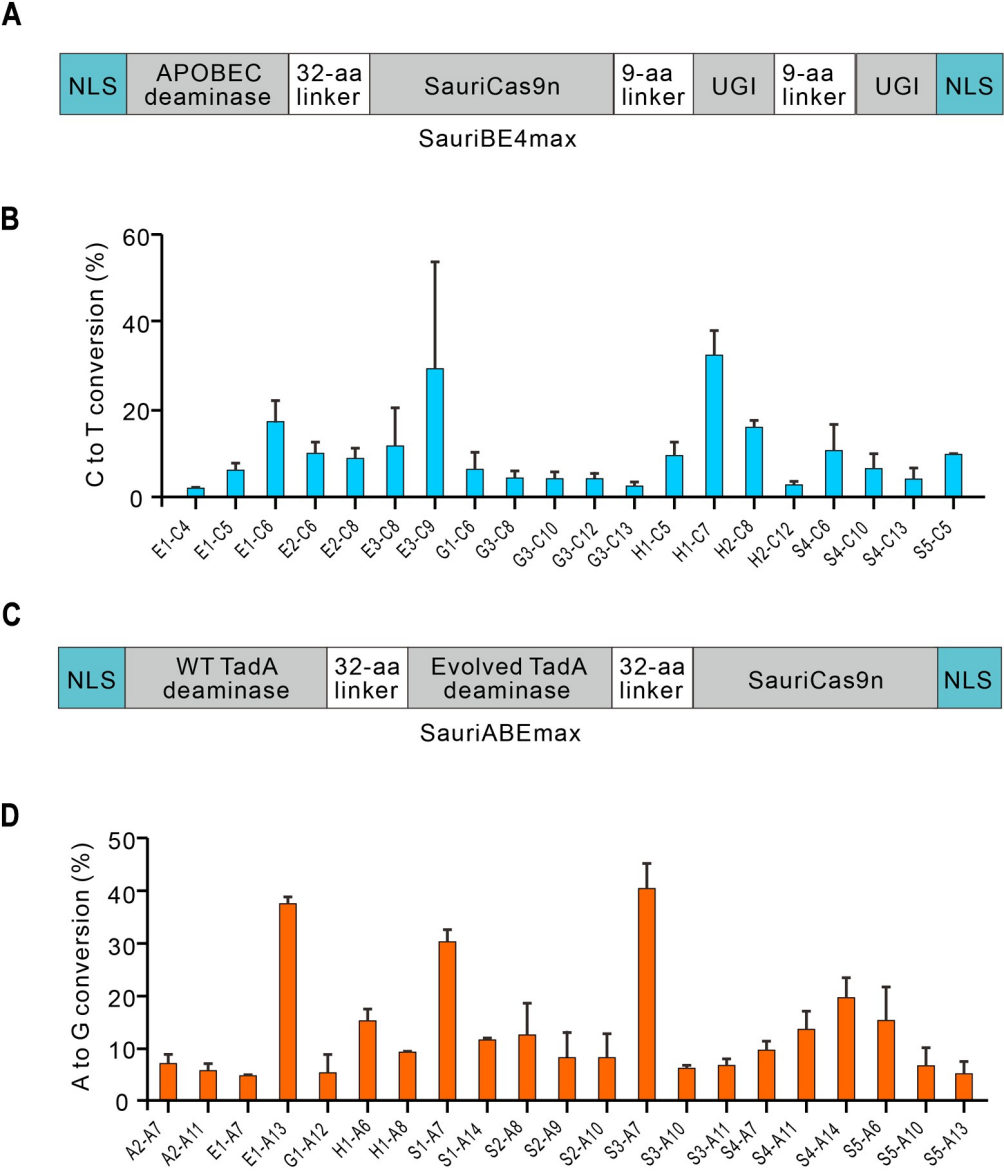
Base editing with SauriCas9. (A) Schematic of the SauriBE4max. (B) SauriBE4max induces C-to-T conversions for a panel of 9 genomic loci. Underlying data for all summary statistics can be found in S1 Data. “E1-C4” means C at target E1 position 4. (C) Schematic of the SauriABEmax. (D) SauriABEmax induces A-to-G conversions for a panel of 9 genomic loci (*n =* 2). Underlying data for all summary statistics can be found in S1 Data. “A2-A7” means A at target A2 position 7. aa, amino acid; GFP, green fluorescent protein; NLS, nuclear localization signal; SauriABEmax, TadA-TadA*(involved TadA)–SauriCas9n; SauriBE4max, APOBEC1–SauriCas9n–UGI; SauriCas9, Cas9 nuclease from *S*. *auricularis*; SauriCas9n, nickase form of SauriCas9.

We replaced the nickase form of SpCas9 with SauriCas9n in BE4max [27] to generate APOBEC1–SauriCas9n–UGI (SauriBE4max, Fig 4A). We transfected HEK293T cells with plasmids encoding SauriBE4max and gRNAs targeting a panel of 9 human genomic loci. After 3 days, targeted deep sequencing revealed that C-to-T base editing occurred (Fig 4B). We also replaced the nickase form of SpCas9 with SauriCas9n in ABEmax [27] to generate TadA-TadA*(involved TadA)–SauriCas9n (SauriABEmax) (Fig 4C). We transfected HEK293T cells with plasmids encoding SauriABEmax and gRNAs targeting a panel of 9 human genomic loci. After 3 days’ editing, targeted deep sequencing revealed that A-to-G base editing occurred (Fig 4D). Collectively, these data demonstrate that SauriCas9 can be used for base editing.

### Specificity analysis of SauriCas9

**Fig 5:**
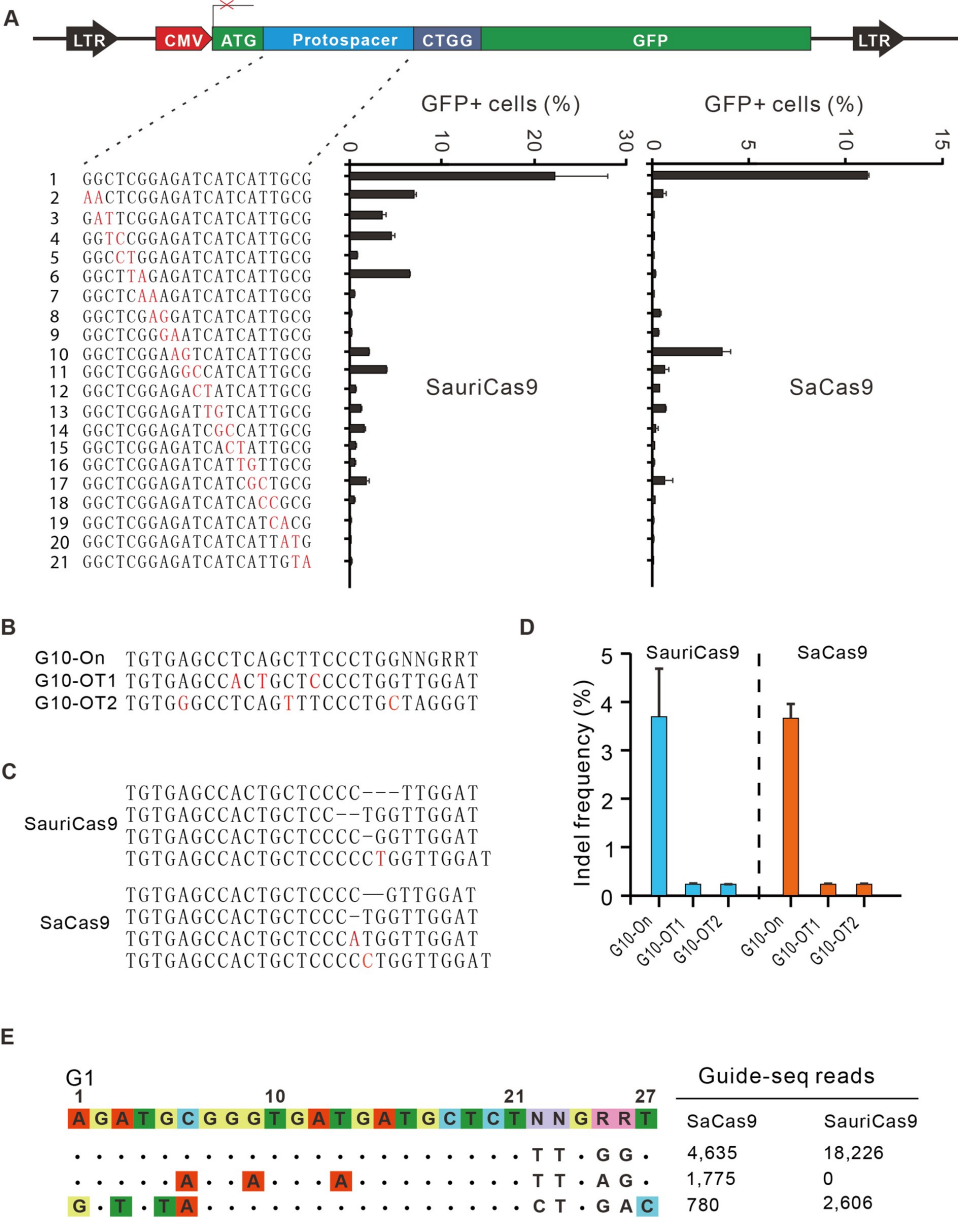
Analysis of SauriCas9 specificity. (A) A target sequence is inserted between ATG and GFP coding sequence, disrupting GFP expression. Target cleavage will induce GFP expression. Underlying data for all summary statistics can be found in S1 Data. A panel of gRNAs with dinucleotide mismatches (red) and each gRNA activity are shown below. (B) Two potential off-target sequences are selected for targeted deep-sequencing analysis. Mismatches are shown in red. (C) Indel sequences are detected by deep sequencing. (D) Indel frequencies detected by targeted deep sequencing (*n =* 2). Underlying data for all summary statistics can be found in S1 Data. (E) Off-targets for G1 locus are analyzed by GUIDE-seq. Read numbers for on- and off-targets are shown on the right. Mismatches compared with the on-target site are shown and highlighted in color. GFP, green fluorescent protein; gRNA, guide RNA; GUIDE-seq, genome-wide, unbiased identification of double-strand breaks enabled by sequencing; indel, insertion/deletion; SaCas9, *S*. *aureus* Cas9; SauriCas9, Cas9 nuclease from *S*. *auricularis*.

Next, we evaluated the off-target activity of SauriCas9 by using the GFP-reporter cell line (Fig 5A). We generated a panel of gRNAs with dinucleotide mutations (Fig 5A). SauriCas9 showed higher activity for both on-target and off-target cleavage compared with SaCas9 (Fig 5A). One limitation of the GFP-reporter assay is that it could not reveal the real indel frequencies. To analyze whether off-target occurs at endogenous loci, we selected a target (G10) with 2 potential off-target sites (G10-OT1 and G10-OT2), which have 3 mismatches and contain PAMs that can be targeted by both SauriCas9 and SaCas9 (Fig 5B). Following transfections of Cas9 + gRNA plasmids, genomic DNA was extracted for targeted deep sequencing. The sequencing results revealed that SauriCas9 and SaCas9 induced indels with very low efficiencies for both off-target sites (Fig 5C and 5D).

To compare genome-wide off-target effects of SauriCas9 and SaCas9, GUIDE-seq assay was performed [28]. Following transfection of Cas9 + gRNA (G1) plasmids and GUIDE-seq oligos, we prepared libraries for deep sequencing. Sequencing and analysis revealed that on-target cleavage occurred for both Cas9 orthologs, reflected by GUIDE-seq read counts (Fig 5E). We identified 2 off-target sites for SaCas9 and 1 off-target site for SauriCas9. Interestingly, SauriCas9 and SaCas9 shared an off-target site because this site contained a PAM for both Cas9 orthologs. In summary, these data indicated that SauriCas9 can induce off-target cleavage at endogenous loci in cells.

### A chimeric Cas9 nuclease displays high fidelity and broad targeting scope

**Fig 6:**
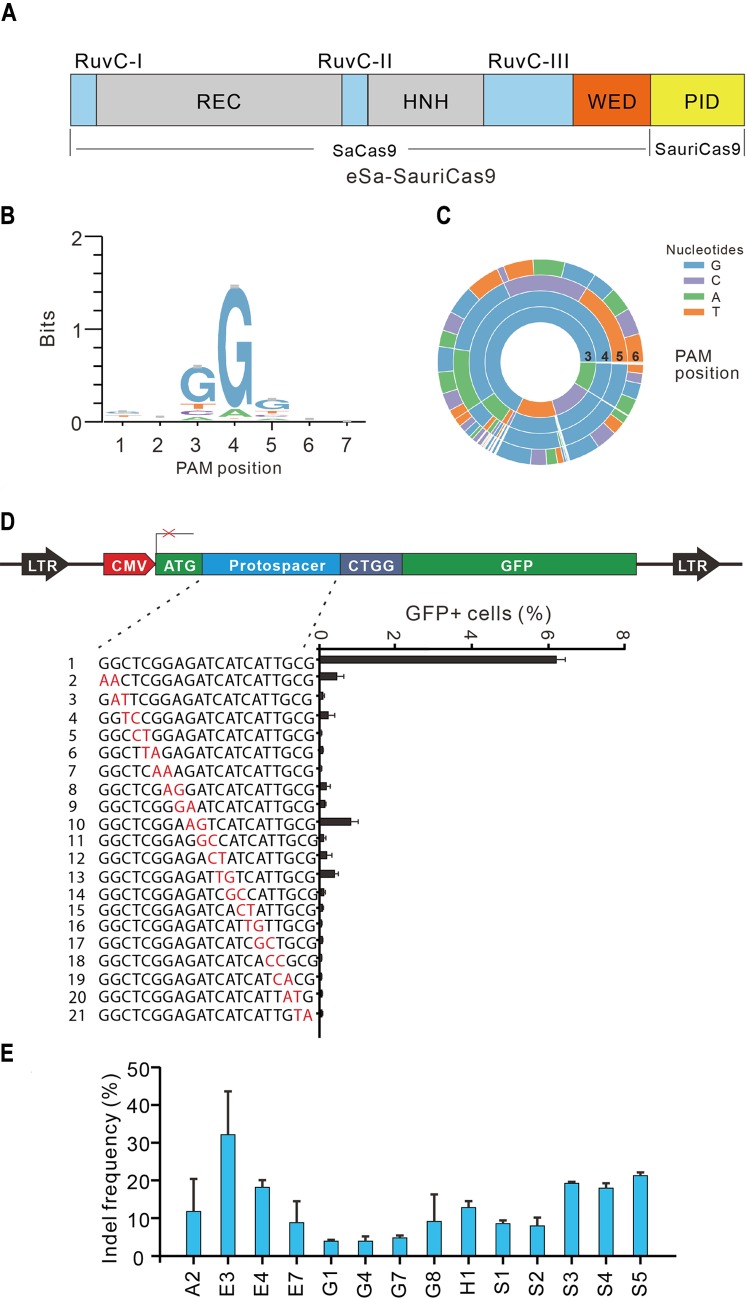
Characterization of chimeric eSa-SauriCas9. (A) Schematic diagram of eSa-SauriCas9. (B-C) WebLogo and PAM wheel of eSa-SauriCas9 are generated from deep-sequencing data. (D) Specificity of eSa-SauriCas9 is measured by the GFP-reporter assay. Underlying data for all summary statistics can be found in S1 Data. A panel of gRNAs with dinucleotide mismatches (red) is shown below. (E) eSa-SauriCas9 generates indels for a panel of 14 endogenous loci (*n* ≥ 2). Underlying data for all summary statistics can be found in S1 Data. eSa-SauriCas9, enhanced specificity SaCas9-SauriCas9; GFP, green fluorescent protein; Indel, insertion/deletion; PAM, protospacer adjacent motif; PID, PAM-interacting domain; SaCas9, a Cas9 derived from *S*. *aureus*; SauriCas9, Cas9 nuclease from *S*. *auricularis*.

Slaymaker and colleagues have generated a high-fidelity version of SaCas9 variant (eSaCas9) by weakening interactions between Cas9 and the target DNA [29]. To generate a Cas9 with high fidelity and broad targeting scope, we replaced the PID of eSaCas9 with that of SauriCas9, resulting in a recombinant chimera, which we named eSa-SauriCas9 (Fig 6A). The GFP-reporter assay revealed that eSa-SauriCas9 recognized NNGG PAM (Fig 6B and 6C) while displaying improved specificity compared with SauriCas9 (Fig 6D). We tested the genome editing capability of eSa-SauriCas9 with a panel of 14 endogenous gene targets in HEK293T cells. eSa-SauriCas9 generated indels at all 14 endogenous loci with varied efficiencies depending on the targets (Fig 6E).

### Expanding SauriCas9 targeting scope

**Fig 7:**
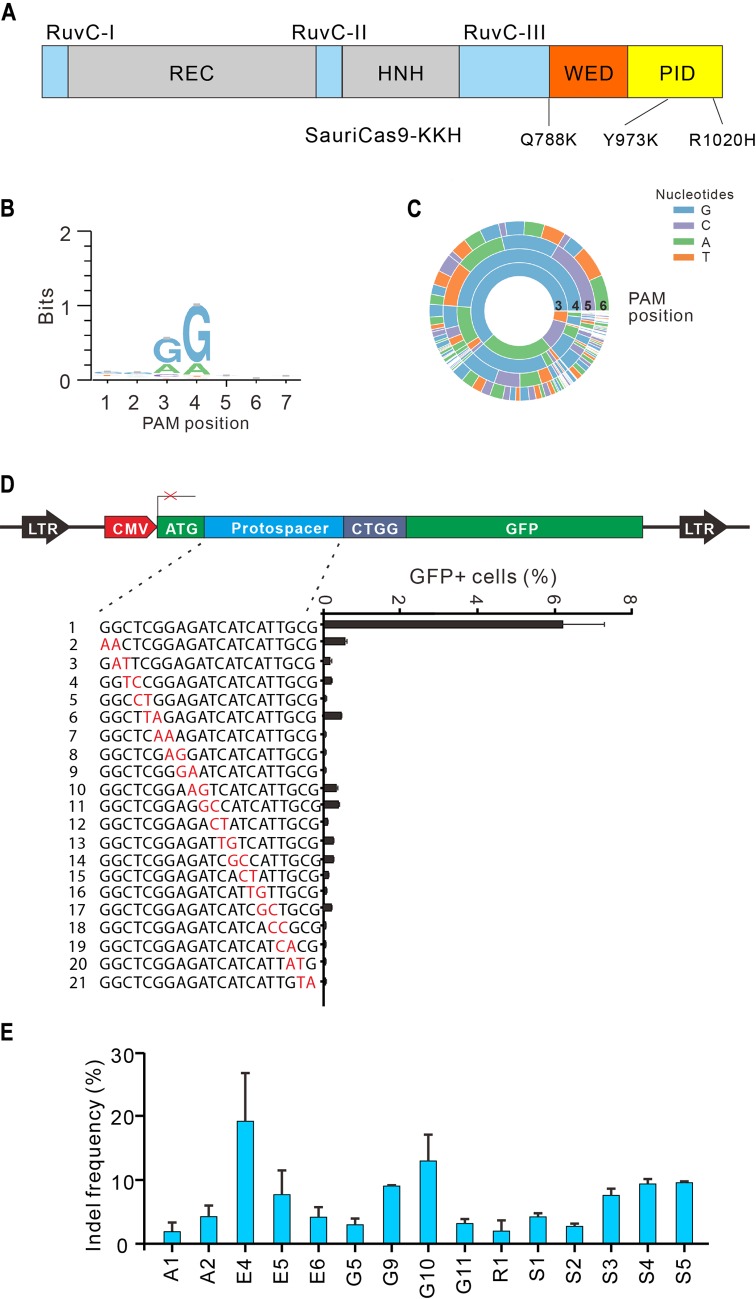
Characterization of SauriCas9-KKH. (A) Schematic diagram of SauriCas9. Q788K/Y973K/R1020H mutations are shown below. (B-C) WebLogo and PAM wheel of SauriCas9-KKH are generated from deep-sequencing data. (D) Specificity of SauriCas9-KKH is measured by the GFP-reporter assay. Underlying data for all summary statistics can be found in S1 Data. A panel of gRNAs with dinucleotide mismatches (red) is shown below. (E) SauriCas9-KKH generates indels for a panel of 15 endogenous loci (*n* ≥ 2). Underlying data for all summary statistics can be found in S1 Data. GFP, green fluorescent protein; gRNA, guide RNA; Indel, insertion/deletion; PAM, protospacer adjacent motif; PID, PAM-interacting domain; SauriCas9, Cas9 nuclease from *S*. *auricularis*; SauriCas9-KKH, triple mutations (Q788K/Y973K/R1020H) on SauriCas9.

A previous study has shown that triple mutations (E782K/N968K/R1015H) on SaCas9 (SaCas9-KKH) relieve restriction on position 3 of PAM, leading to NNNRRT recognition [30]. To broaden the targeting scope of SauriCas9, we introduced triple mutations (Q788K/Y973K/R1020H) that are identical to E782K/N968K/R1015H on SaCas9 (S1A Fig), resulting in SauriCas9-KKH (Fig 7A). GFP-reporter assay revealed that SauriCas9-KKH preferred NNRG PAM (Fig 7B and 7C). We inserted a target with NNAG PAM into a GFP-reporter plasmid and compared the efficacy between SauriCas9 and SauriCas9-KKH on this PAM. The results revealed that SauriCas9-KKH was more effective with NNAG PAM (S7 Fig). The GFP-reporter assay revealed that SauriCas9-KKH displayed improved specificity compared with SauriCas9 overall (Fig 7D). We tested the editing capacity of SauriCas9-KKH with a panel of 15 endogenous targets, and indels could be easily detected after genome editing (Fig 7E).

## Discussion

Small Cas9 nucleases (<1,100 aas) that can be packaged into an AAV hold great promise for gene therapy. Although several small Cas9 nucleases have been used for genome editing, the range of targetable sequences remains limited because of the requirement for a PAM sequence flanking a given target site. SaCas9 is the first small Cas9 ortholog that has been delivered by AAV vector for in vivo genome editing [8], but this Cas9 ortholog is used infrequently because of the requirement for a longer PAM sequence (NNGRRT). Engineered SaCas9 variants can increase targeting scope [30,31], but this increase in targeting scope often comes at a cost of reduced on-target activity. Three types of II-C Cas9 orthologs have been used for mammalian genome editing, including *N*. *meningitidis* Cas9 (NmeCas9) [10,13], *Campylobacter jejuni* Cas9 (CjeCas9) [11] and *N*. *meningitidis* Cas9 (Nme2Cas9) [9]. These 3 Cas9 nucleases recognize N4GAYW/N4GYTT/N4GTCT, N4RYAC, and N4CC PAMs, respectively. However, type II-C Cas9 nucleases generally display low editing efficiency [12,14]. More recently, CasX (<1,000 aas) recognizing TTCN PAM has been shown to enable genome editing in mammalian cells [32], expanding the targeting scope of small Cas nucleases.

By using a GFP-reporter strategy that we previously developed [16], we identified SauriCas9 as a novel nuclease for mammalian genome editing. SauriCas9 consists of 1,061 aas, which can be packaged into AAV for genome editing. Importantly, SauriCas9 recognizes a simple NNGG PAM, expanding the targeting scope of small Cas9 nucleases. We also generated a SauriCas9-KKH to expand the targeting scope. In addition, we have demonstrated the versatility of SauriCas9, which can be adapted for base editing. Recently, mini-SaCas9 that only retains DNA binding activity has been developed [33,34]. Mini-SaCas9 can be used for a variety of applications by fusing with other effectors. It will be interesting to engineer SauriCas9 for mini-SauriCas9 in the future. With further development, we anticipate that SauriCas9 can be an important genome editing tool for both basic research and clinical applications.

## Materials and methods

### Cell culture and transfection

HEK293T cells were maintained in Dulbecco’s Modified Eagle Medium (DMEM) supplemented with 10% FBS (Gibco), 100 U/mL penicillin, and 100 mg/mL streptomycin at 37°C and 5% CO_2_. For SauriCas9 PAM sequence screening, HEK293T cells were plated in 10-cm dishes and transfected at approximately 60% confluency with SauriCas9-gRNA-expressing plasmid (15 μg) using Lipofectamine 2000 (Life Technologies). For genome editing and base editing capability of SauriCas9, HEK293T cells were seeded on 48-well plates and transfected with SauriCas9+gRNA or SauriABEmax/SauriBE4max+gRNA plasmid (500 ng) using Lipofectamine 2000 (Life Technologies) in Opti-MEM (Gibco) according to the manufacturer’s instructions.

### Plasmid construction

#### Cas9 expression plasmid construction

Vector backbone of pX601 (Addgene #61591) was used to express Cas9. pX601 plasmid was PCR amplified using primers pX601-F/pX601-R to remove SaCas9; human codon–optimized Cas9 gene was obtained from Addgene (S1 Table) except SauriCas9, SauriCas9-KKH, eSa- SauriCas9, which were synthesized by HuaGene (Shanghai, China); each Cas9 gene was cloned into pX601 backbone by NEBuilder Assembly Tool (NEB) following the manufacturer’s instructions, resulting in AAV-CMV-Cas9. Sequences were verified by Sanger sequencing (GENEWIZ, Suzhou, China).

#### AAV-CMV-SauriCas9-puro

The plasmid of AAV-CMV-SauriCas9 was linearized by restriction enzyme BamH1 (NEB). The gene that expresses puromycin was PCR amplified from PX459 (Addgene #118632) using primers Gibson-puro-F/Gibson-puro-R and then cloned into AAV-CMV-SauriCas9 backbone by NEBuilder Assembly Tool (NEB) to generate AAV-CMV-SauriCas9-puro plasmid.

#### SauriBE4max and SauriABEmax

pCMV_ABEmax_P2A_GFP (Addgene #112101) and pCMV_AncBE4max (Addgene #112094) plasmids were linearized by PCR amplification using primers ABEmax-F/ABEmax-R and AncBE4max-F/ AncBE4max-R to remove SpCas9n, respectively. SauriCas9n was generated by PCR amplification from pAAV-CMV-SauriCas9 plasmid with primers ABESauriCas9(D15A)-F/ABE-SauriCas9(D15A)-R and BE4-SauriCas9(D15A)-F/ BE4-SauriCas9 (D15A)-R, respectively. The PCR products were cloned into linearized pCMV-ABEmax_P2A_GFP and pCMV_AncBE4max backbone to generate SauriABEmax and SauriBE4max, respectively.

#### hU6-Sa_tracr plasmid

The pX601 vector containing hU6-Sa_tracr was PCR amplified using primer hU6-F/ORI-R, followed by phosphorylation with T4 polynucleotide kinase (NEB) and religation with T4 DNA ligase (NEB). gRNAs were inserted into hU6-Sa_tracr plasmid between 2 Bsa1 restriction sites. All primer sequences are listed in S2 Table; all target sequences are listed in S3 Table.

### PAM sequence analysis

Twenty base pair sequences (AAGCCTTGTTTGCCACCATG/GTGAGCAAGGGCG AGGAGCT) flanking the target sequence (GAACGGCTCGGAGATCATCATTGCG NNNNNNN) were used to fix the target sequence. GCG and GTGAGCAAGGGCG AGGAGCT were used to fix 7-bp random sequence. Target sequences with in-frame mutations were used for PAM analysis. The 7-bp random sequence was extracted and visualized by WebLog3 [35] and PAM wheel chart to demonstrate PAMs [36].

### Verification of PAM sequence with GFP-reporter constructs

Two plasmids containing NNGG (CTGG) and NNNGG (TCTGG) PAM sequences were isolated from the PAM library. Each plasmid was packaged into lentivirus to generate stable cell lines. To remove background mutations that induce GFP expression, the GFP-negative cells were sorted by MoFlo XDP machine. The sorted cells were seeded into 24 wells and transfected with SauriCas9+gRNA plasmid (800 ng) by Lipofectamine 2000 (Life Technologies). Three days after editing, the GFP-positive cells were analyzed on the Calibur instrument (BD). Data were analyzed using FlowJo.

### Genome editing of SauriCas9 at endogenous sites

HEK293T cells were seeded into 24 wells and transfected plasmids expressing Cas9 nucleases (500 ng) together with plasmids expressing gRNA (300 ng) by Lipofectamine 2000 (Life Technologies). Cells were collected 3 days after transfection. For HeLa, A375, A459, HFF, and N2a cells, we transfected AAV-CMV-SauriCas9-puro (500 ng) and hU6-Sa_tracr-gRNA (300 ng) by Lipofectamine 3000 (Life Technologies). Cells were selected by puromycin and collected 5 to 7 days after transfection. Genomic DNA was isolated, and the target sites were PCR amplified by nested PCR amplification and purified by a Gel Extraction Kit (QIAGEN) for deep sequencing.

### Base editing with SauriABEmax/SauriBE4max

HEK293T cells were seeded into 24 wells and transfected with SauriABEmax/SauriBE4max and hU6-Sa_tracr-gRNA. Cells were collected, and the genomic DNA was isolated 3 days after transfection. The target sites were PCR amplified and extracted by a Gel Extraction Kit (QIAGEN). The efficiency of the base editing was measured by deep sequencing.

### Test of SauriCas9 specificity

To test the specificity of SauriCas9, we generated a GFP-reporter cell line with NNGG (CTGG) PAM. The cells were seeded into 48 wells and transfected with pAAV-CMV-SauriCas9/pAAV-CMV-SaCas9 (300 ng) and hU6-Sa_gRNA (200 ng) by Lipofectamine 2000 (Life Technologies). Three days after editing, the GFP-positive cells were analyzed on a Calibur instrument (BD). Data were analyzed using FlowJo.

### Test of SauriCas9 specificity

To test the specificity of SauriCas9, we generated a GFP-reporter cell line with NNGG (CTGG) PAM. The cells were seeded into 48 wells and transfected with Cas9-expressing plasmids (300 ng) and hU6-Sa_gRNA plasmids (200 ng) by Lipofectamine 2000 (Life Technologies). Three days after editing, the GFP-positive cells were analyzed on a Calibur instrument (BD). Data were analyzed using FlowJo.

### AAVs production

HEK293T cells were seeded at approximately 40% confluency in a 6-cm dish the day before transfection. For each well, 2 μg of Cas9/gRNA expressing plasmid, 2 μg of pAAV-RC (GeneBank: AF369963), and 4 μg of pAAV-helper were transfected using 80 μl of PEI (0.1% m/v, Polysciences, Cat# 23966 [pH 4.5]). Media was changed 8 hours after transfection. After 72 hours, cells are scrapped and poured into a 15-mL conical centrifuge tube. Spin at 3,000 rpm at 4°C for 10 minutes, and transfer supernatant into a new 15-mL tube. Resuspend the cell pellet in 1 mL of RB TMS Buffer (50 mM Tris-HCl, 150 mM NaCl [pH 8.0]). Transfer to a new 15-mL conical tube. Freeze in dry ice–ethanol bath for 10 minutes, and thaw at 37°C for 10 minutes, and repeat 3 times. Spin at 3,000 rpm at 4°C for 10 minutes. Mix the 2 supernatants together and filter with a 0.45-μm polyvinylidene fluoride filter. Add one-half volume of the mixed solution (1 M NaCl + 10% PEG8000), and incubate at 4°C overnight. After centrifugation at 4°C for 2 hours at 12,000 rpm, discard the flow-through, and add 200 μL of chilled RB TMS and add the flow-through into a 12 well with approximately 80% confluency HEK293T.

### GUIDE-seq

GUIDE-seq experiments were performed as described previously [28], with minor modifications. Briefly, 2×10^5^ HEK293T cells were transfected with 1 μg of AAV-CMV-SauriCas9/Px601 (AAV-CMV-SaCas9), 0.5 μg of hU6-Sa_tracr-gRNA plasmid, and 100 pmol of annealed GUIDE-seq oligonucleotides by electroporation and then seeded into 12 wells. Electroporation voltage, width, and number of pulses were 1,150 V, 30 ms, and 1 pulse, respectively. Genomic DNA was extracted with a DNeasy Blood and Tissue kit (QIAGEN) 5 days after transfection according to the manufacturer’s protocol. Library preparation and sequencing were performed exactly as described previously [28].

### Quantification and statistical analysis

All the data are shown as mean ± SD. Statistical analyses were conducted using Microsoft Excel. Two-tailed, paired Student’s *t* tests were used to determine statistical significance when comparing 2 groups. A value of *p* < 0.05 was considered to be statistically significant. (*p < 0.05, **p < 0.01, ***p < 0.001).

## Supporting information

S1 Fig Protein sequence alignment for SauriCas9 and SaCas9. D15 on SauriCas9 is indicated by green box; Q788, Y973, and R1020 on SauriCas9 are indicated by light blue box; S991 and L996 on SauriCas9 are indicated by red box; PID sequences are underlined. PID, protospacer adjacent motif–interacting domain; SaCas9, a Cas9 derived from *S*. *aureus*; SauriCas9, a Cas9 derived from *S*. *auricularis*.
(TIF)

S2 Fig qRT-PCT analysis of SaCas9 and SauriCas9 expression (*n =* 3). Underlying data for all summary statistics can be found in S1 Data. SaCas9, a Cas9 derived from *S*. *aureus*; SauriCas9, a Cas9 derived from *S*. *auricularis*.
(TIF)

S3 Fig SauriCas9 enables genome editing in diverse cell types. (A) Genome editing for a panel of 6 loci in A375, A549, HFF, and HeLa cells. Underlying data for all summary statistics can be found in S1 Data. (B-E) Genome editing for additional loci in A375, A549, HFF, HeLa, and N2a cells (*n* ≥ 2). Underlying data for all summary statistics can be found in S1 Data. HFF, human foreskin fibroblast; SauriCas9, a Cas9 derived from *S*. *auricularis*.
(TIF)

S4 Fig Genome editing of SauriCas9 at NNNGG PAM is inefficient (*n =* 2). Underlying data for all summary statistics can be found in S1 Data. PAM, protospacer adjacent motif; SauriCas9, a Cas9 derived from *S*. *auricularis*.
(TIF)

S5 Fig SauriCas9 can be delivered by AAV for genome editing. (A) Genome editing for a panel of 8 loci in HEK293T, HCT116, HFF, and A375 cells. Underlying data for all summary statistics can be found in S1 Data. (B-E) Genome editing for additional loci in HEK293T, HCT116, HFF, and A375 cells (*n* ≥ 2). Underlying data for all summary statistics can be found in S1 Data. AAV, adeno-associated virus; HFF, human foreskin fibroblast; SauriCas9, a Cas9 derived from *S*. *auricularis*.
(TIF)

S6 Fig SauriCas9n can induce nicks on genomic DNA. (A) Schematic diagram of experimental design. A pair of target sequences (underlined) is inserted into GFP coding sequence (green), leading to inactivation of GFP. Genome editing will result in expression of a portion of GFP due to in-frame mutations. PAM is indicated by red; triangles indicate cleavage sites. (B) Transfection of SauriCas9 or SauriCas9n with gRNAs results in GFP expression. gRNA, guide RNA; PAM, protospacer adjacent motif; SauriCas9, a Cas9 derived from *S*. *auricularis*; SauriCas9n, nickase form of SauriCas9.
(TIF)

S7 Fig GFP-reporter assay reveals that SauriCas9-KKH is more efficient than SauriCas9 with NNAG PAM. PAM, protospacer adjacent motif; SauriCas9, a Cas9 derived from *S*. *auricularis*; SauriCas9-KKH, triple mutations (Q788K/Y973K/R1020H) on SauriCas9.
(TIF)

S1 Data Underlying values for all reported summary statistics. Raw data from all reported summary statistics.
(XLSX)

S1 Table tracrRNA sequence and human codon–optimized Cas9 gene. The Cas9 gene of SauriCas9, SauriCas9-KKH, and eSa-SauriCas9 were synthesized; others were obtained from Addgene. eSa-SauriCas9, enhanced specificity SaCas9-SauriCas9; SaCas9, a Cas9 derived from S. aureus; SauriCas9, a Cas9 derived from *S*. *auricularis*; SauriCas9-KKH, triple mutations (Q788K/Y973K/R1020H) on SauriCas9.
(XLSX)

S2 Table Primers used in this study. List of oligonucleotide pairs and primer used for deep sequencing and plasmid construction.
(XLSX)

S3 Table Target sites used in this study. List of the endogenous target site of human and mouse and their downstream PAM. PAM, protospacer adjacent motif.
(XLSX)

## Abbreviations

aa: amino acid
ABE: adenine base editor
AAV: adeno-associated virus
CBE: cytosine base editor
CjeCas9: *Campylobacter jejuni* Cas9
DSB: double-strand break
GFP: green fluorescent protein
eSaCas9: a high-fidelity version of SaCas9 variant
eSa-SauriCas9: enhanced specificity SaCas9-SauriCas9
gRNA: guide RNA
GUIDE-seq: genome-wide, unbiased identification of DSBs enabled by sequencing
HFF: human foreskin fibroblast
indel: insertion/deletion
NmeCas9: *Neisseria meningitidis* Cas9
Nme2Cas9: *Neisseria meningitidis* Cas9
PAM: protospacer adjacent motif
PID: PAM-interacting domain
qRT-PCR: quantitative reverse transcription polymerase chain reaction
SaCas9: *Staphylococcus aureus* Cas9
SauriABEmax: TadA-TadA*(involved TadA)–SauriCas9n
SauriBE4max: APOBEC1–SauriCas9n–UGI
SauriCas9: Cas9 nuclease from *Staphylococcus auricularis*
SauriCas9n: nickase form of SauriCas9
SauriCas9-KKH: triple mutations (Q788K/Y973K/R1020H) on SauriCas9
SpCas9: Cas9 derived from *Streptococcus pyogenes*
tracrRNA: trans-activating CRISPR RNA
