# Supplementary figures and images for: A compact Cas9 ortholog from Staphylococcus Auricularis (SauriCas9) expands the DNA targeting scope
Source: PLoS Biol. 2020 Mar 30;18(3):e3000686. doi: 10.1371/journal.pbio.3000686 (PMC7145270; doi:10.1371/journal.pbio.3000686)

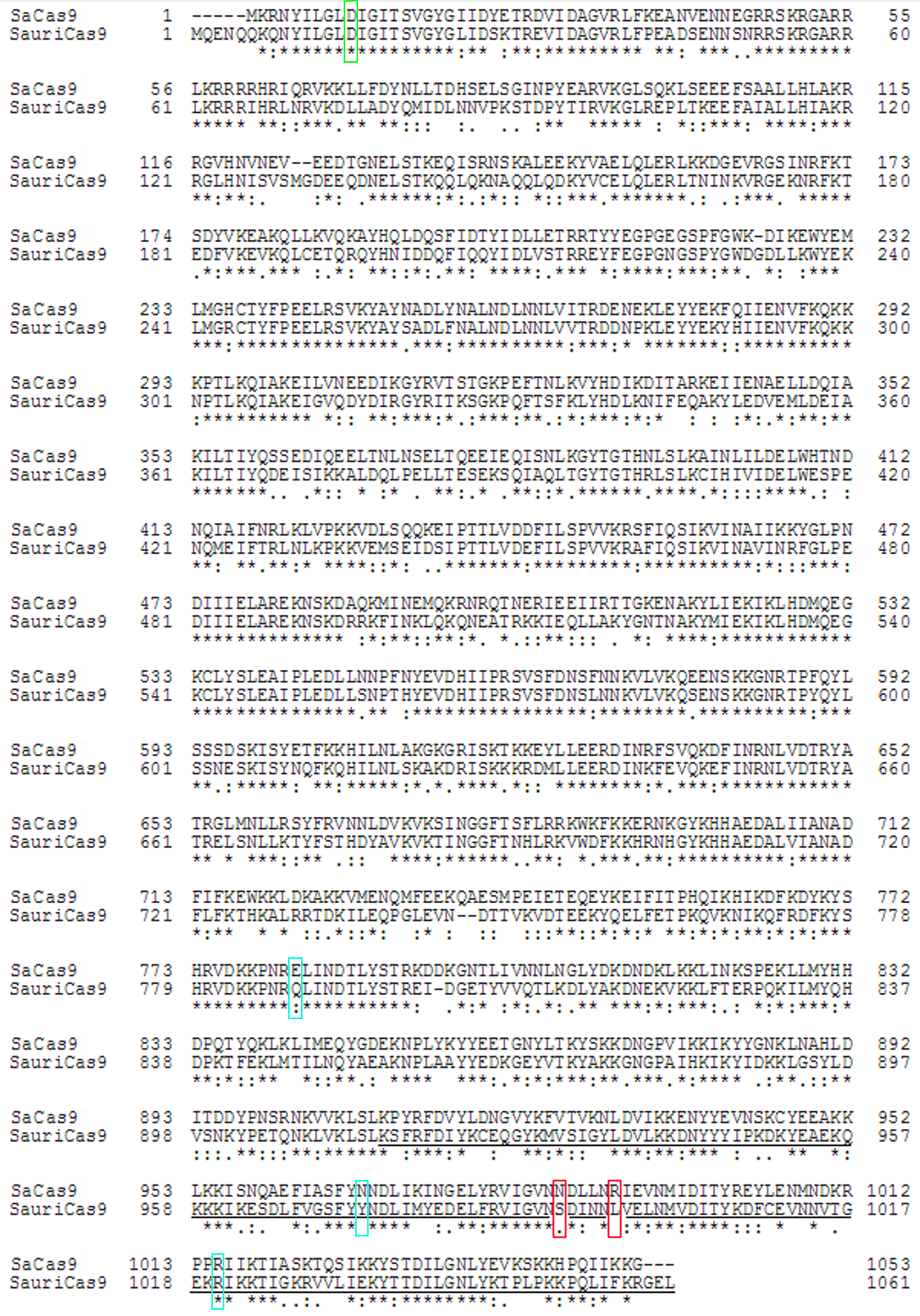

Supplement: S1 Fig — D15 on SauriCas9 is indicated by green box; Q788, Y973, and R1020 on SauriCas9 are indicated by light blue box; S991 and L996 on SauriCas9 are indicated by red box; PID sequences are underlined. PID, protospacer adjacent motif–interacting domain; SaCas9, a Cas9 derived from S. aureus; SauriCas9, a Cas9 derived from S. auricularis. (TIF) [file pbio.3000686.s001.tif]

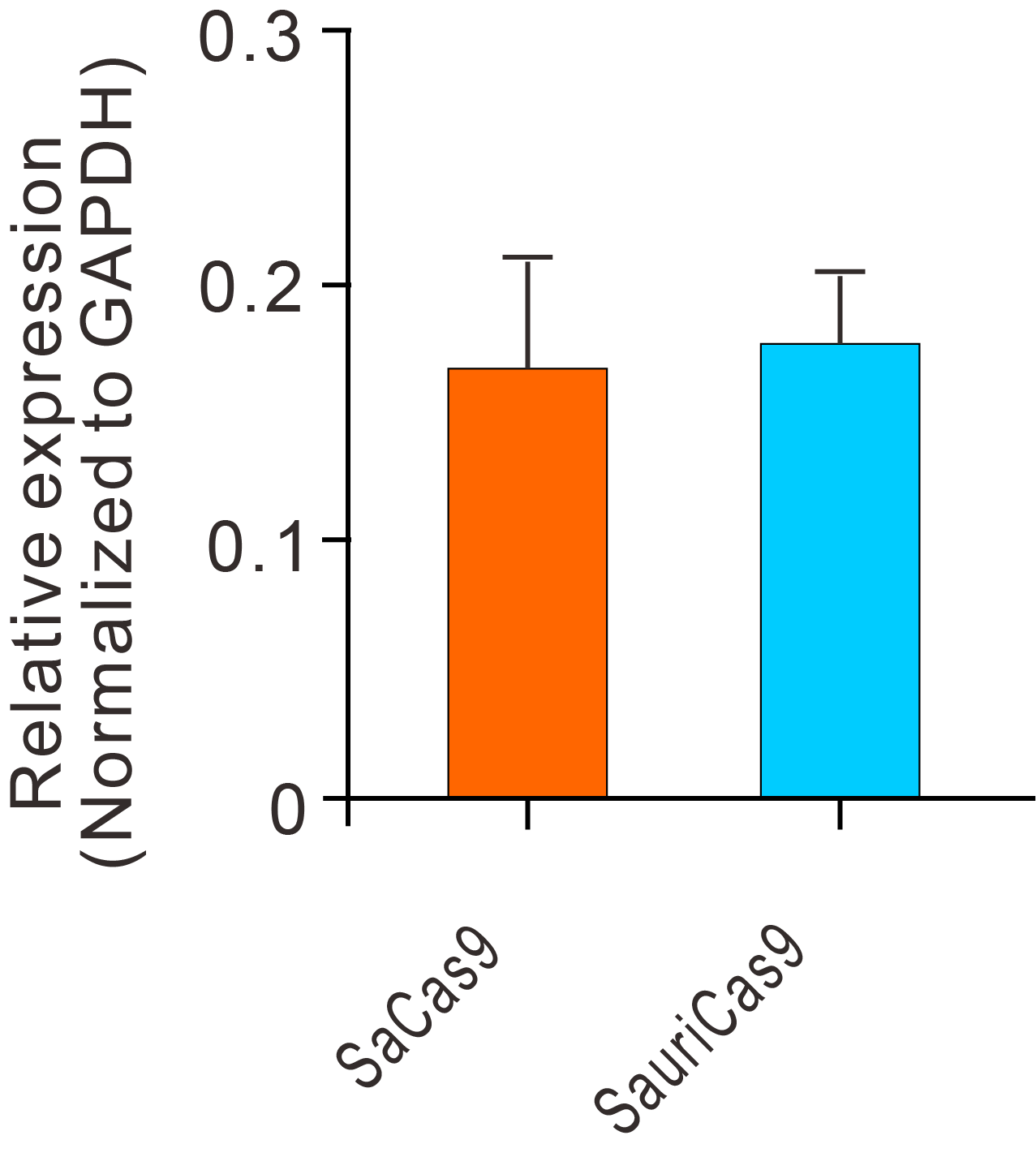

Supplement: S2 Fig — Underlying data for all summary statistics can be found in S1 Data. SaCas9, a Cas9 derived from S. aureus; SauriCas9, a Cas9 derived from S. auricularis. (TIF) [file pbio.3000686.s002.tif]

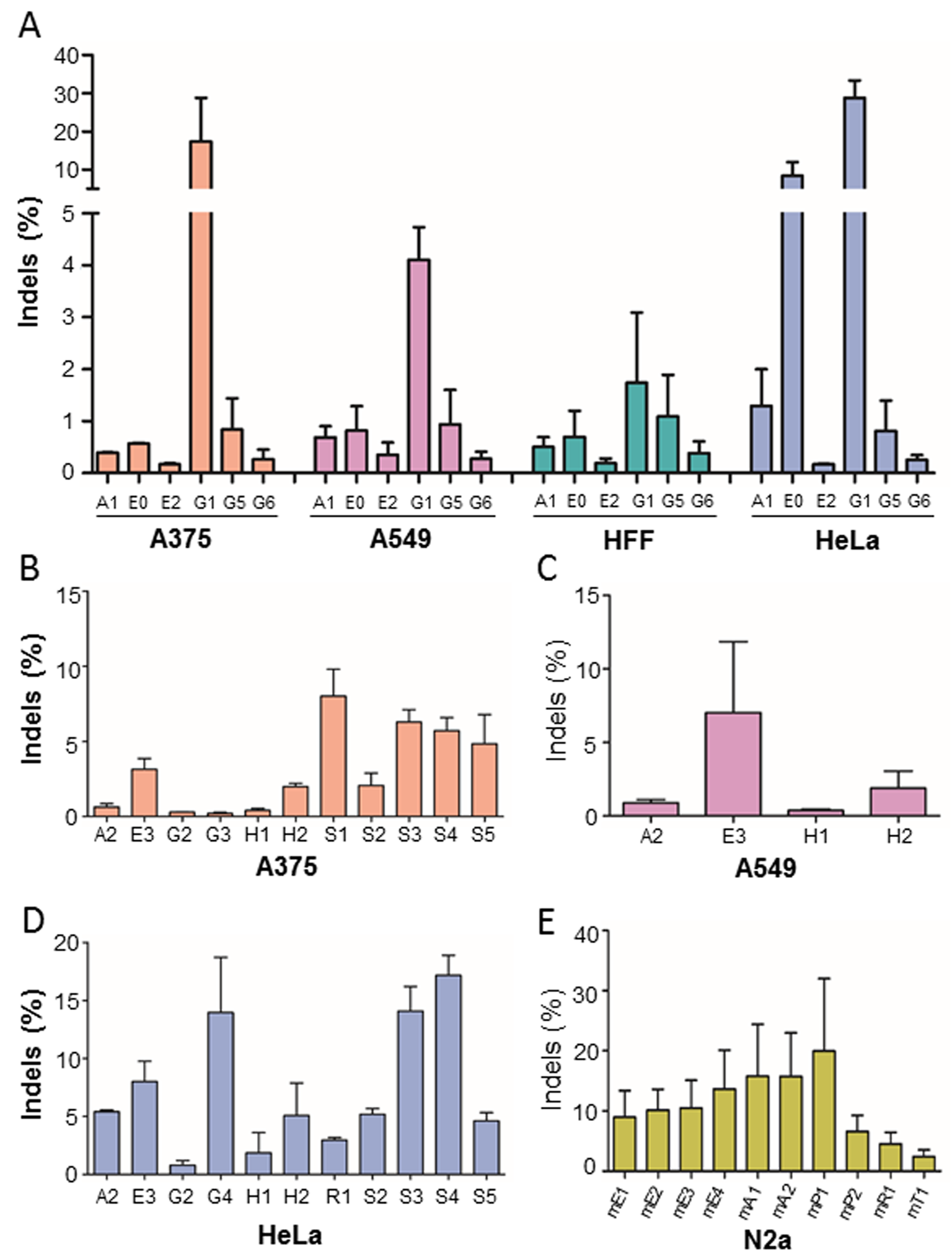

Supplement: S3 Fig — (A) Genome editing for a panel of 6 loci in A375, A549, HFF, and HeLa cells. Underlying data for all summary statistics can be found in S1 Data. (B-E) Genome editing for additional loci in A375, A549, HFF, HeLa, and N2a cells (n ≥ 2). Underlying data for all summary statistics can be found in S1 Data. HFF, human foreskin fibroblast; SauriCas9, a Cas9 derived from S. auricularis. (TIF) [file pbio.3000686.s003.tif]

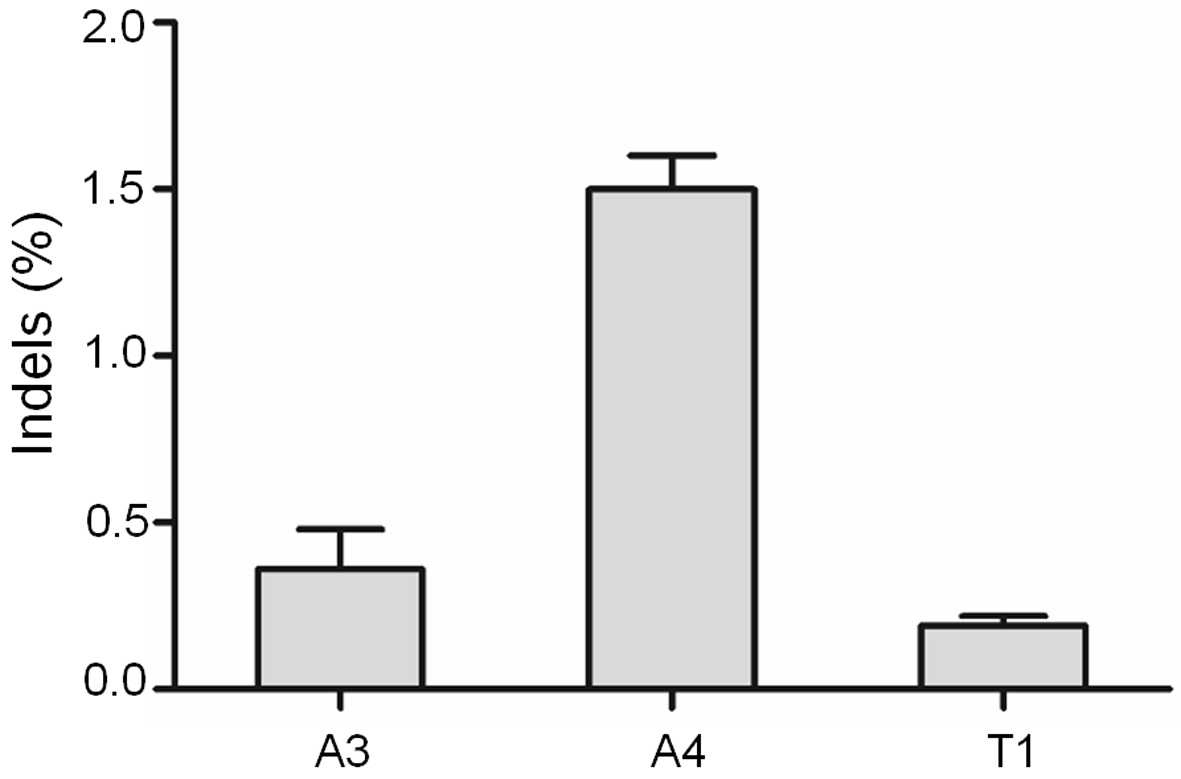

Supplement: S4 Fig — Underlying data for all summary statistics can be found in S1 Data. PAM, protospacer adjacent motif; SauriCas9, a Cas9 derived from S. auricularis. (TIF) [file pbio.3000686.s004.tif]

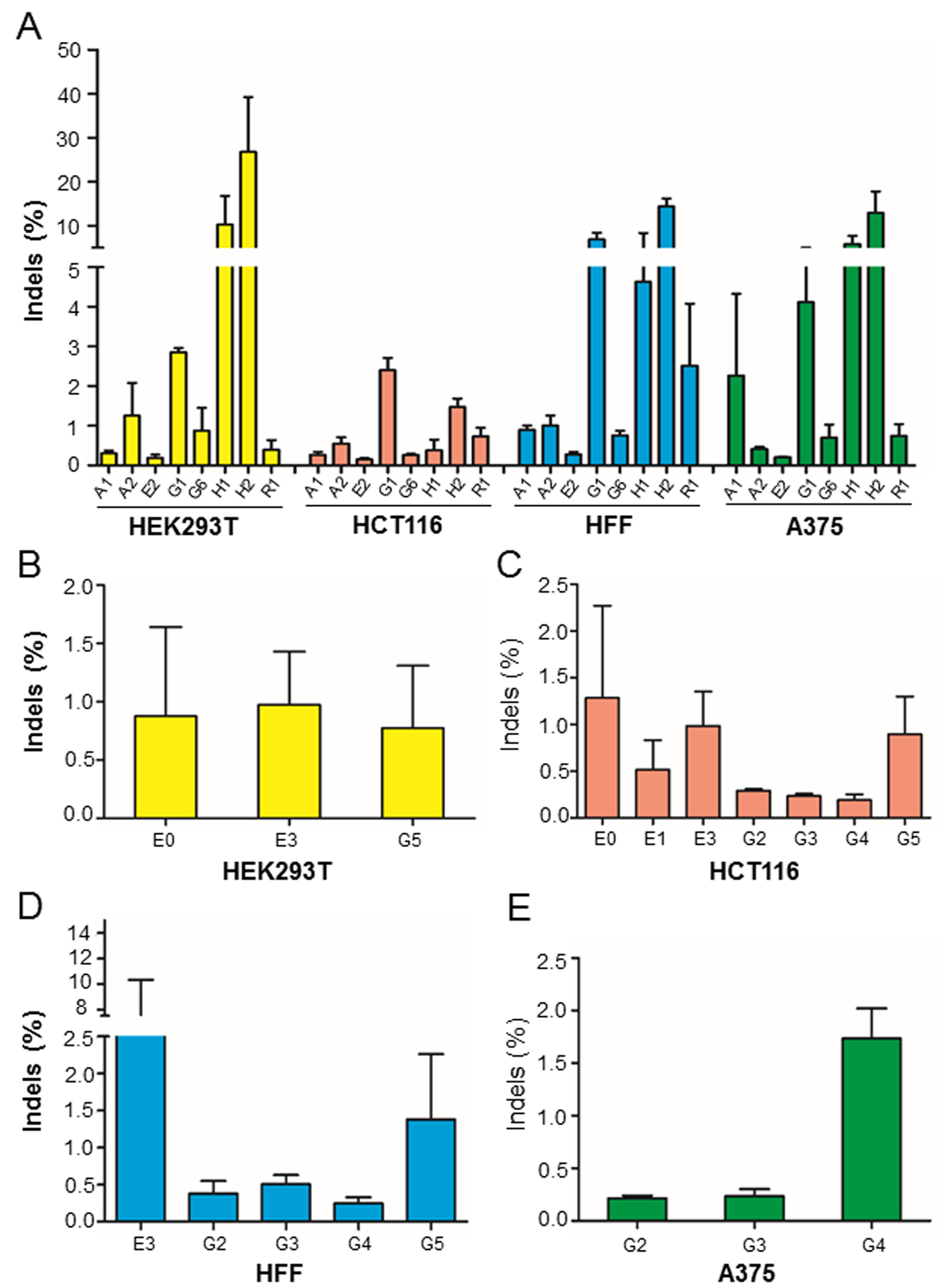

Supplement: S5 Fig — (A) Genome editing for a panel of 8 loci in HEK293T, HCT116, HFF, and A375 cells. Underlying data for all summary statistics can be found in S1 Data. (B-E) Genome editing for additional loci in HEK293T, HCT116, HFF, and A375 cells (n ≥ 2). Underlying data for all summary statistics can be found in S1 Data. AAV, adeno-associated virus; HFF, human foreskin fibroblast; SauriCas9, a Cas9 derived from S. auricularis. (TIF) [file pbio.3000686.s005.tif]

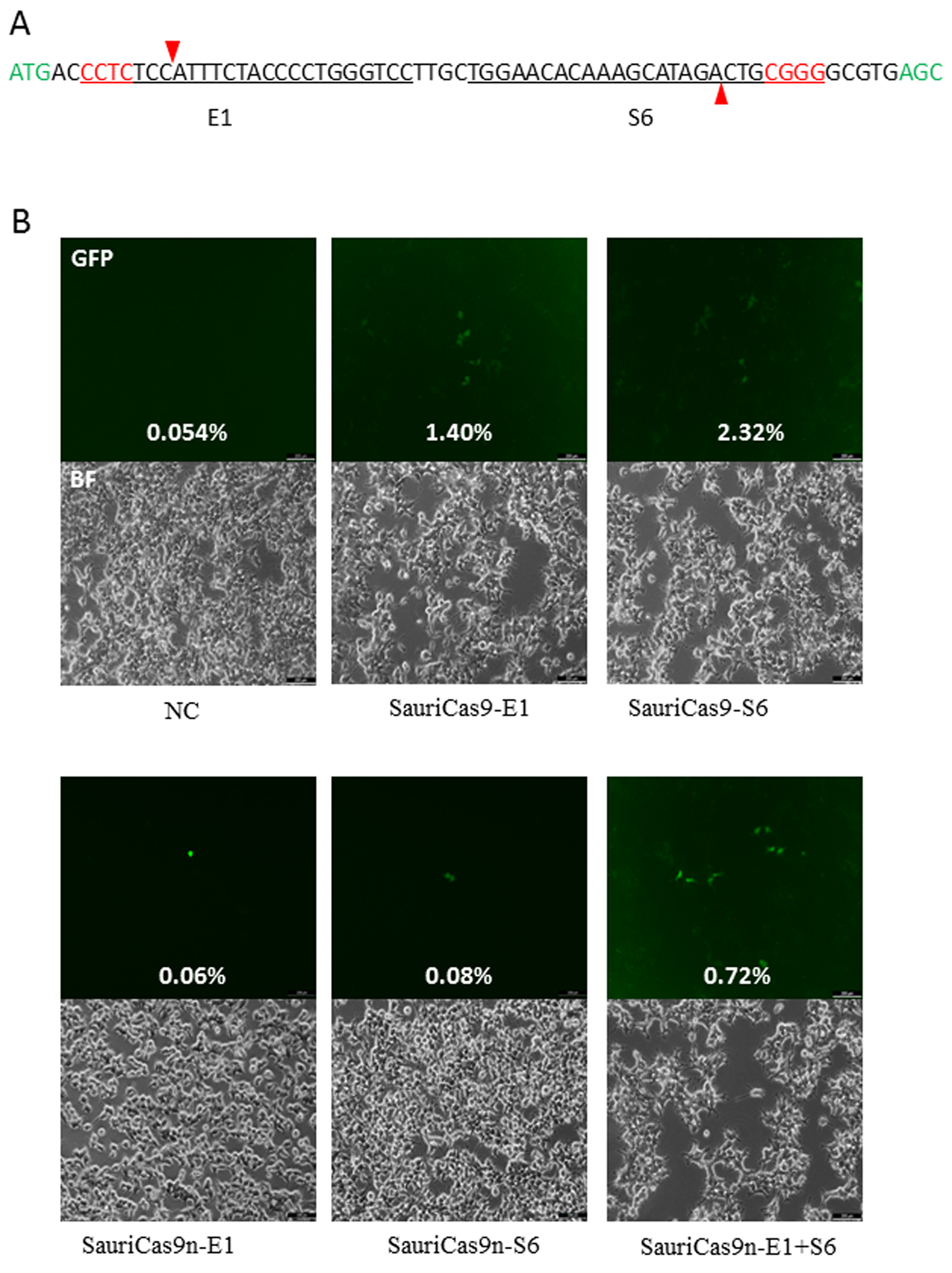

Supplement: S6 Fig — (A) Schematic diagram of experimental design. A pair of target sequences (underlined) is inserted into GFP coding sequence (green), leading to inactivation of GFP. Genome editing will result in expression of a portion of GFP due to in-frame mutations. PAM is indicated by red; triangles indicate cleavage sites. (B) Transfection of SauriCas9 or SauriCas9n with gRNAs results in GFP expression. gRNA, guide RNA; PAM, protospacer adjacent motif; SauriCas9, a Cas9 derived from S. auricularis; SauriCas9n, nickase form of SauriCas9. (TIF) [file pbio.3000686.s006.tif]

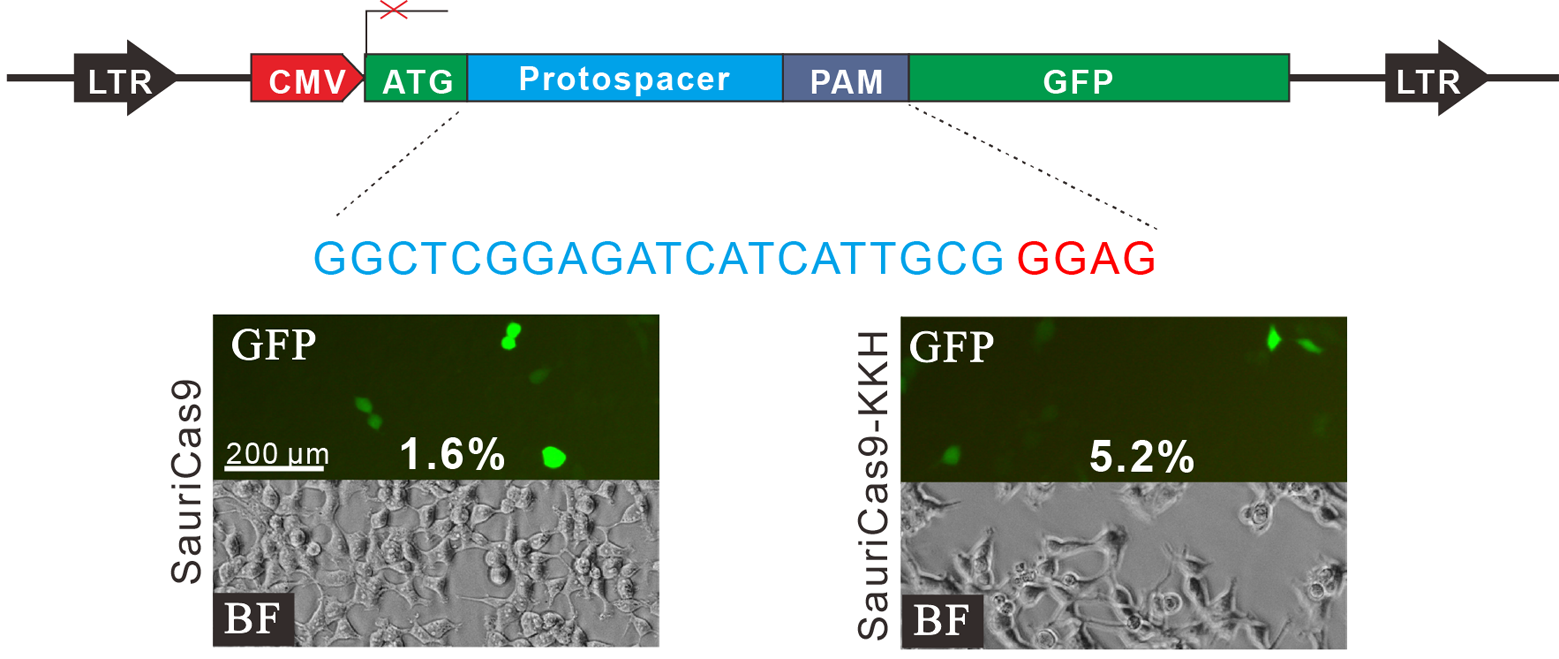

Supplement: S7 Fig — PAM, protospacer adjacent motif; SauriCas9, a Cas9 derived from S. auricularis; SauriCas9-KKH, triple mutations (Q788K/Y973K/R1020H) on SauriCas9. (TIF) [file pbio.3000686.s007.tif]
